# Platelet Surface-Associated Activation and Secretion-Mediated Inhibition of Coagulation Factor XII

**DOI:** 10.1371/journal.pone.0116665

**Published:** 2015-02-17

**Authors:** Natalia V. Zakharova, Elena O. Artemenko, Nadezhda A. Podoplelova, Anastasia N. Sveshnikova, Irina A. Demina, Fazly I. Ataullakhanov, Mikhail A. Panteleev

**Affiliations:** 1 National Research Center for Hematology, Moscow, Russia; 2 Center for Theoretical Problems of Physicochemical Pharmacology, Moscow, Russia; 3 Federal Research and Clinical Center of Pediatric Hematology, Oncology and Immunology, Moscow, Russia; 4 Faculty of Physics, M.V. Lomonosov Moscow State University, Moscow, Russia; 5 Faculty of Biological and Medical Physics, Moscow Institute of Physics and Technology, Dolgoprudny, Russia; University of Kentucky, UNITED STATES

## Abstract

Coagulation factor XII (fXII) is important for arterial thrombosis, but its physiological activation mechanisms are unclear. In this study, we elucidated the role of platelets and platelet-derived material in fXII activation. FXII activation was only observed upon potent platelet stimulation (with thrombin, collagen-related peptide, or calcium ionophore, but not ADP) accompanied by phosphatidylserine exposure and was localised to the platelet surface. Platelets from three patients with grey platelet syndrome did not activate fXII, which suggests that platelet-associated fXII-activating material might be released from α-granules. FXII was preferentially bound by phosphotidylserine-positive platelets and annexin V abrogated platelet-dependent fXII activation; however, artificial phosphotidylserine/phosphatidylcholine microvesicles did not support fXII activation under the conditions herein. Confocal microscopy using DAPI as a poly-phosphate marker did not reveal poly-phosphates associated with an activated platelet surface. Experimental data for fXII activation indicates an auto-inhibition mechanism (*k*_i_/*k*_a_ = 180 molecules/platelet). Unlike surface-associated fXII activation, platelet secretion inhibited activated fXII (fXIIa), particularly due to a released C1-inhibitor. Platelet surface-associated fXIIa formation triggered contact pathway-dependent clotting in recalcified plasma. Computer modelling suggests that fXIIa inactivation was greatly decreased in thrombi under high blood flow due to inhibitor washout. Combined, the surface-associated fXII activation and its inhibition in solution herein may be regarded as a flow-sensitive regulator that can shift the balance between surface-associated clotting and plasma-dependent inhibition, which may explain the role of fXII at high shear and why fXII is important for thrombosis but negligible in haemostasis.

## Introduction

Coagulation factor XII (fXII) is a serine protease zymogen that can undergo auto-activation in the presence of foreign surfaces. FXII is the main component of contact blood clotting initiation. However, fXII deficiency in humans and animals is not associated with a tendency for bleeding, which indicates that fXII is dispensable for haemostasis [1]; however, it has been implicated in other physiological processes [2]. The significance of fXII is only undisputed under certain conditions, such as implant-associated coagulation, or for in vitro assays, such as the activated partial thromboplastin time assay.

Interest in fXII recently increased due to the findings that fXII-deficient mice were protected from thrombosis [3], [4], [5] and that fXIIa inhibitors prevent arterial thrombosis without affecting haemostasis in mice [6] and primates [7]. These observations suggest that the role of fXIIa must be reconsidered, which has stimulated research on (patho)physiological fXII activation mechanisms. Platelets are the major arterial thrombi components and are considered potential fXII activators. As early as fifty years ago, studies indicated that stimulated platelets can accelerate fXII activation, at least in plasma [8], [9], and this hypothesis was confirmed in a recent study, which demonstrated that platelets in plasma can bind fXII and accelerate its activation [10]. However, reports demonstrate that platelet-secreted polyphosphates (poly-P), platelet-derived microparticles (MPs) and circulating plasma microparticles are alternative potential fXII activators [11], [12], [13]. In contrast, more recent studies claim that the platelet-derived poly-P chain length is insufficient for efficient fXII activation [14], and the overall role of platelets as well as platelet-derived poly-P in fXII activation has been questioned [15] and debated [16], [17]. A mechanism for fXIIa involvement in thrombus formation has not been proposed to explain its dispensability in haemostasis; further, no study has analysed the potential roles for platelet subpopulations [18], [19], [20], which differentially bind other coagulation factors [21].

Given such discrepancies, we attempted to study fXII activation and answer the following questions. i) Can platelets activate fXII or simply accelerate its activation using other plasma proteins? ii) Does the platelet surface directly participate in fXII activation? iii) Do platelet subpopulations differ in fXII binding and activation? iv) Finally, which platelet component is most powerful for fXII activation: the platelet surface, MPs or released materials (including poly-P)?

The results herein demonstrate preferential fXII binding by procoagulant phosphatidylserine (PS)-exposing platelets, which promote fXII activation through their surface, retain bound essential levels of fXIIa, and trigger contact pathway clotting in recalcified plasma regardless of the material secreted outside the platelet. Because ‘grey’ platelets (from grey platelet syndrome patient blood) cannot activate fXII, α-granule components are likely important for developing the fXII-activating capacity. Under the conditions herein, the platelet surface was the most powerful fXII activating component compared with platelet-derived microparticles and material secreted outside the platelet. Unexpectedly, the full effect of the material secreted outside the platelet was inhibitory, not activating. The C1-inhibitor (C1-INH) was released and specifically inhibited FXIIa. Based on the results and earlier published data, we constructed a computer model that demonstrates a potential role for filtration velocity in regulating thrombi contact pathway activation.

## Materials and Methods

### Materials

The following materials were used: thrombin (Haematologic Technologies; Essex Junction, VT, USA); fXIIa and fXII (Enzyme Research Laboratories; South Bend, IN, USA); AlignFlow flow cytometry alignment beads (2.5 μm for 488-nm excitation), fluorescein-5-isothiocyanate (FITC), and tetramethylrhodamine (TMRM) (Molecular Probes; Eugene, OR, USA); unlabelled and FITC-annexin V (BD Biosciences; San Jose, CA, USA); AlexaFluor-647-annexin V (Biolegend; San Diego, CA, USA); prostaglandin E1 (PGE1) (MP Biochemicals; Irvine, CA, USA); PPACK (EMD Chemicals; Gibbstown, NJ, USA); the chromomeric substrates S2238 and S2302 (Chromogenix; Milano, Italy); HEPES, bovine serum albumin, Sepharose CL-2B, Protein G sepharose, apyrase grade VII, mepacrine (quinacrine), PBS, EDTA and DMSO (Sigma-Aldrich; St Louis, MO, USA); calpeptin and MDL 28170 (Tocris Bioscience; Ellisville, MO, USA); G1/C1-inhibitor and polyclonal goat anti-human serpin G1/C1-inhibitor antibody (R&D Systems; Minneapolis, MN, USA); goat polyclonal anti-human factor XII antibody (LifeSpan BioSciences, Inc., Seattle, WA, USA); peroxidase-AffiniPure donkey anti-goat IgG (Jackson ImmunoResearch Laboratories; West Grove, PA, USA); egg phosphatidylcholine and egg phosphatidylserine (Avanti Polar Lipids; Alabaster, AL, USA); DAPI (4′,6-Diamidino-2-phenylindole dihydrochloride) (AppliChem; Darmstadt, Germany); and a kit to estimate fXII activation (Renam; Moscow, Russia). Collagen-related peptide (CRP) was kindly provided by Prof. R.W. Farndale (University of Cambridge, Cambridge, UK), and the FXIIa inhibitor corn trypsin inhibitor (CTI)) was kindly provided by Dr. Smolyaninov (Institute of Protein Research, Russian Academy of Sciences, Pushchino, Russia). Active site thrombin was titrated using PPACK and S2238 as previously described [22]. Tissue factor (TF) inhibitor fVIIai was generated through fVIIa inactivation as previously described [23].

### Ethics statement

All blood collection procedures were performed in accordance with the Declaration of Helsinki using a protocol approved by the institution's Ethics Committee of the Center for Theoretical Problems of Physicochemical Pharmacology, National Research Center for Hematology, and Federal Research and Clinical Center of Pediatric Hematology, Oncology and Immunology. Written informed consent was obtained from all donors and patients.

### Blood collection, platelet isolation and plasma preparation

Blood was collected, added to 106 mM sodium citrate (9:1) and supplemented with apyrase (0.1 U/ml) and PGE1 (1 μM). After precipitation from platelet-rich plasma (PRP), platelets were resuspended in buffer A (150 mM NaCl, 2.7 mM KCl, 1 mM MgCl_2_, 0.4 mM NaH_2_PO_4_, 20 mM HEPES, 5 mM glucose, and 0.5% bovine serum albumin, pH 7.4) and purified using gel-filtration as previously described [24]. To prepare chelated plasma for an fXII activation assay, the plasma was further centrifuged (after platelet precipitation) for 30 min at 16,000 g to remove the MPs [25], diluted to 40% with buffer A, and supplemented with 8 mM sodium EDTA (pH 7.6). Calcium chelation was necessary to exclude thrombin generation and fXIIa-independent S2302 cleavage. For recalcification, 90% MP-depleted plasma containing 10.6 mM was supplemented with 12.5 mM CaCl_2_ immediately prior to the assay. The specific fXIIa inhibitor corn trypsin inhibitor (CTI) was used at 200 mg/mL (S1A Fig.); the level of its effect did not depend on the timing of its addition (i.e., either simultaneously with platelet addition or 15 min thereafter) (S1B Fig.). The specific TF antagonist fVIIai was used at 50 nM; its potency was verified in a TF-dependent clotting assay (S1C Fig.).

### Patients with grey platelet syndrome

Three patients with grey platelet syndrome (GPS) were members of the same family and were involved in the study herein: a 92-year-old woman (GPS-1), her 66-year-old son (GPS-2), and his 35-year-old son (GPS-3). All of the subjects exhibited characteristic laboratory and clinical features of GPS: severe α-granule protein deficiencies; large, agranular, and grey platelets; moderate thrombocytopenia (131–173(10^9^/L); recurrent ecchymosis; and epistaxis. GPS-2 and GPS-3 platelets exhibited lower aggregation upon stimulation with ADP (30% and 34%, respectively). The numbers of major adhesion receptors (glycoprotein Ib and glycoprotein IIb-IIIa), integrin activation, dense granule release (using a mepacrine assay), and phosphatidylserine exposure were normal for all patients.

### Platelet activation, separation of MPs and the platelet secretion, and secretion removal

The platelets were activated at 2×10^8^/mL for 15 min using 10 nM thrombin, 10 μg/mL CRP, 10 μM A23187 or 100 μM 2-MeS-ADP in the presence of 2.5 mM CaCl_2_. Non-activated platelets, which were used as a control, were incubated for 15 min with 2.5 mM CaCl_2_. Activation was stopped through 50–100-fold dilution in buffer A (for thrombin, 100 nM PPACK was added first). To separate the MPs and platelet secretion, the activated platelets were diluted to 4×10^6^/mL and centrifuged at 300 g for platelet precipitation [25]. The supernatant containing the platelet secretion and MPs was further centrifuged at 16,000 g to precipitate the MPs [25].

For the experiments in which the platelet secretion was removed, A23187-activated platelets were washed through double precipitation at 16,000 g for 10 min. A23187 stimulation typically yields approximately 90% of PS-positive and poorly aggregated platelets, which facilitates secretion removal through platelet precipitation.

### Factor XII labelling and binding experiments

FXII (3.3 mg/ml) was dialysed, supplemented with 0.1 M sodium bicarbonate (pH 9.0) as well as 10 mg/ml FITC (MR = 5), and continuously stirred for 2 h (4°C). The reaction was stopped through a 30-min incubation with 1.5 M hydroxylamine (pH 8.5). To separate the conjugate, the mixture was dialysed against PBS (1 h). The level of labelling was spectrophotometrically controlled. Estimates of degraded protein (using SDS-PAGE, S1D Fig.) and the FITC-fXII activation capacity (using a fXII activity kit) confirmed that ≥90% of the total protein corresponded to a non-activated factor. For the binding assay, activated platelets (100 nM thrombin) were incubated for 5 min with FITC-fXII (0–1000 nM) and AlexaFluor 647-annexin V (1.5% v/v), diluted 10-fold, and immediately analysed. The fluorescence intensity was converted to the mean number of molecules/platelet using a calibration curve obtained using the Green Flow Cytometry Intensity Calibration Kit at 488-nm excitation and 515-nm emission (Invitrogen).

### Flow cytometry characterisation of platelet subpopulations and MPs

Activated platelets were incubated for 3 min with annexin V (1–5% vol/vol), diluted 20-fold, and analysed using a FACSCalibur (BD Biosciences, San Jose, CA, USA) or Accuri C6 (Accuri Cytometers, Ann Arbor, MI, USA) flow cytometer. The MPs were determined as annexin V-positive events with a lower characteristic FSC than platelets (as shown in S2 Fig.). The number of events was controlled using calibration beads. The data were processed using WinMDI 2.8 (Joseph Trotter, Scripps Research Institute, La Jolla, CA, USA) or CFlow (Accuri Cytometers) software.

### Platelet-dependent fXIIa generation

Platelet-dependent fXIIa generation was measured as an increase in optical density at 405 nm due to chromogenic substrate S2302 cleavage using a Multiskan Ascent microplate reader (Thermo Scientific, Hudson, NH, USA). For experiments using purified fXII, 100 μL of activated platelets (2×10^6^/mL), 5 μL S2302 (4 mM) and 2 μL fXII at the indicated concentrations were mixed in a 96-well microtitre plate. For the plasma-based assay, 50 μL of platelets (4×10^6^/mL) were mixed with 5 μL S2302 and 50 μL of 40% plasma. The reaction mixtures were incubated for 5–10 min at room temperature to generate a steady-state equilibrium between fXIIa formation and its auto-inhibition [26] under the conditions herein. Next, the steady-state fXIIa activity was measured at 37°C. For the controls, the platelets were replaced with buffer A containing equivalent platelet activator concentrations (finally diluted 100-fold). To account for optical density decreases due to concurrent platelet aggregation, another control reaction was performed using platelets without S2302. A typical calculation for the resulting platelet-dependent reaction is shown in S3A Fig. In control experiments, fXIIa formation was not inhibited by S2302 (S3B Fig.). The first-order curves confirmed that the platelet-dependent, steady-state fXIIa concentration did not increase during measurement. The results are generally expressed as steady-state concentrations of the fXIIa formed, which were calculated from the slopes using a correlation coefficient obtained from purified fXIIa (S3C Fig.).

### Confocal microscopy

Glass coverslips (24×24 mm, Heinz Herenz, Hamburg, Germany) were cleaned with potassium dichromate, coated with 20 mg/ml fibrinogen (buffer A, 40 min, room temperature) and used for flow-chamber assembly. The platelets (1×10^8^/mL) were pre-incubated with 50 μM DAPI (as a poly-P marker [27]), 200 nM TMRM (mitochondria marker) and/or 10 μM mepacrine (dense granule marker) at 37°C (for 1 h, 15 min, and 30 min, respectively); introduced into the flow-chamber; and allowed to spread on fibrinogen for 10 min. The unattached platelets were then washed-out using buffer A, and the fibrinogen-attached platelets were applied for a 15-min activation event using 100 nM thrombin with 2.5 mM CaCl_2_. Next, the activator was washed-out using buffer A (containing 2.5 mM CaCl_2_ or 1 mM EDTA) and replaced with 3% annexin V or 5% anti-fibrinogen antibody (respectively) for 5 minutes at room temperature. After subsequently replacing annexin V or anti-fibrinogen antibody with the corresponding buffer, images were acquired using an Axio Observer.Z1 microscope (Carl Zeiss, Jena, Germany) with a 405 nm excitation laser, 520 nm filter and 100× oil immersion objective. Z-series scanning was performed using 0.31-μm steps through a 5 μm z-depth.

### PS and PC liposome preparation

Lipids were incubated in chloroform:methanol (95:5 vol/vol). Lipid films were prepared through evaporation and hydration to generate large multilamellar vesicles, which, after freeze-thawing, were extruded through 100-nm-pore polycarbonate membranes to produce unilamellar vesicles. PS-liposomes comprised 20% PS and 80% PC, and PC-liposomes comprised 100% PC.

### Western blot analyses of purified and platelet-released C1-INH

After precipitating 2×10^8^/mL non-activated platelets and MPs at 16,000 g for 30 min (containing a portion of the platelet secretion, 5 μg/mL BSA and 2.5 mM CaCl_2_), the supernatant was incubated at 37°C for 30 min with 25 nM fXIIa and/or 20 μM S2302. Next, the total protein was concentrated 20-fold through precipitation with 0.015% Na^+^-deoxycholate and 5% TCA and diluted in 0.1 N NaOH. After reduction with 2% β-mercaptoethanol, the samples were applied to 4–10% gradient SDS-PAGE and electroblotted. Western blotting was performed using goat antihuman C1-INH antibody as the primary antibody and peroxidase-conjugated anti-goat IgG as the second antibody. The resulting bands were developed using the enhanced chemiluminescence (ECL) method, and images (3-min exposure) were collected using the Gel Doc XR+ System (Bio-Rad, Berkeley, CA, USA).

### C1-INH immuno-precipitation

Purified fXII was added into a non-activated platelet suspension (2×10^8^/mL) through 280 nM. The platelets were activated at 37°C for 30 min (10 μM A23187, 2.5 mM CaCl_2_), incubated with goat antihuman C1-INH antibody (1:100 dilution) at room temperature for 1 h, and mixed with Protein G Sepharose (1% vol/vol). After a 4 h incubation at 4°C (under gentle rotation) Protein G Sepharose was precipitated at 200 g for 30 sec, washed 5 times using buffer A (without BSA) at 4°C, mixed with 2 lysis buffer for SDS PAGE (1/20 of the initial suspension volume), and denatured for 5 min at 96°C. The supernatant was applied to SDS PAGE. The control immuno-precipitation experiment was performed using platelets activated without fXII.

### Computer modelling of fXII activation

FXII activation and C1-INH release from a platelet aggregate were simulated using the Virtual Cell environment (http://vcell.org). Based on our experiments, we constructed a computational model of fXII activation that included fXII surface activation and auto-inhibition in a platelet suspension [26]. Next, we constructed an *in silico* aggregate comprising 15 platelets, each of which can activate fXII, using parameters from the “fXII activation” model. The aggregate dimensions were 1012 μm, and the maximum time step was 0.1 s. To ensure simulation accuracy, the simulation results were compared with results generated using a finer mesh size and smaller time steps. The mesh size, maximum time step and tolerances were adjusted as necessary. The models “fXII activation” and “C1 washout” are accessible at the Virtual Cell website vcell.org/vcell_models/published_models.html under share models/agolomy.

### Statistics

The experiments were reproduced at least three times using platelets from different donors. The mean values ±SD were calculated from three parallel measurements for the same, typical donor unless otherwise specified. The non-parametric Mann-Whitney test and Welch’s unpaired t-test were used to estimate the statistical significance. In the figures, the asterisk symbol (*) indicates significantly different results (at a p value <0.05).

## Results

### FXII binding and activation by platelets

**Fig 1:**
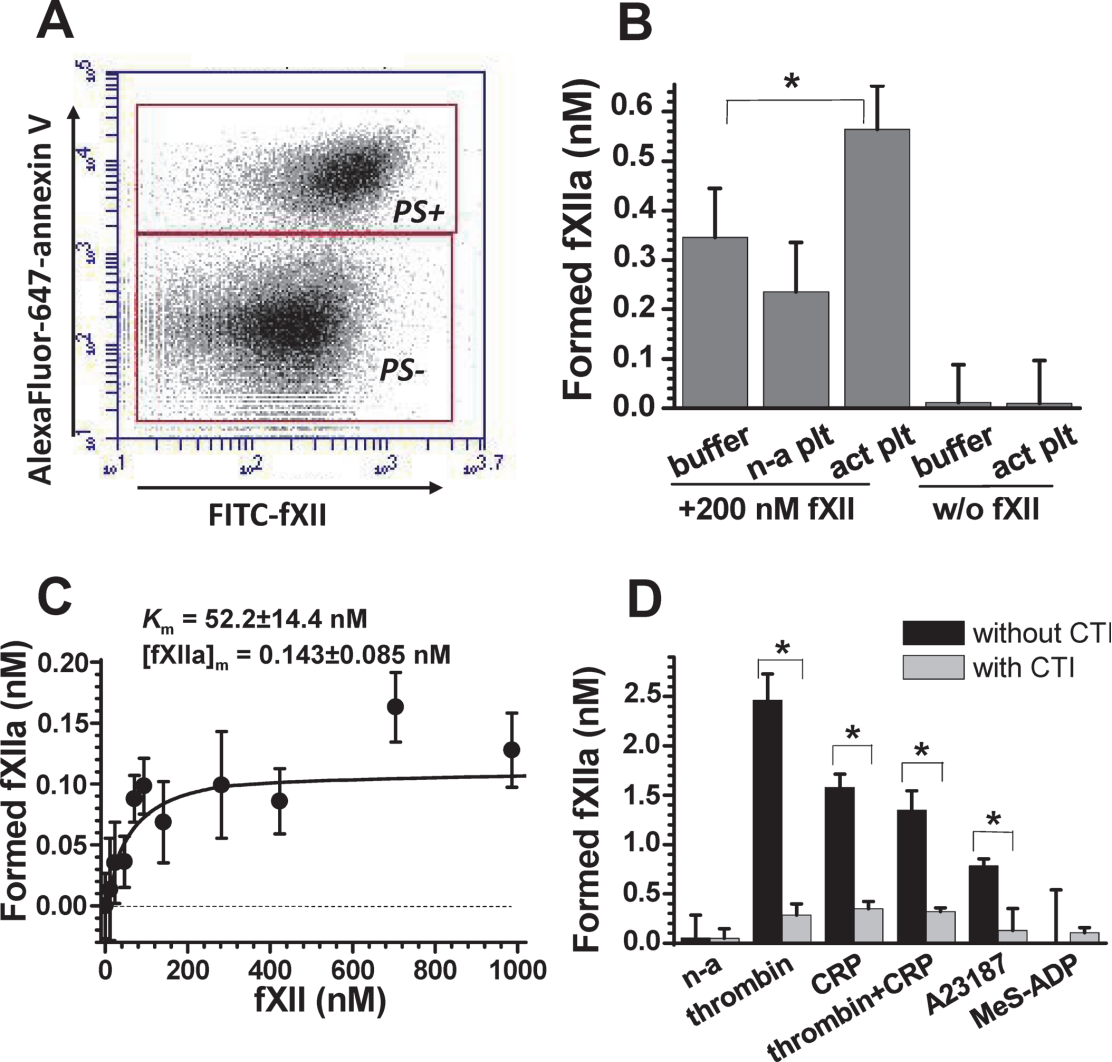
Platelet activation of purified and plasma fXII. **(A)** Flow-cytometry dot-plots demonstrating predominant FITC-fXII binding for PS-positive platelet subpopulations (labelled “PS+”). The result corresponds to 1000 nM FITC-fXII binding. **(B)** The effect of platelets on activation of purified 200 nM fXII. Platelets were activated by 10 nM thrombin (n = 3). **(C)** Dose-dependence for platelet-dependent fXIIa formation upon a reaction between thrombin-activated platelets and purified fXII (n = 3). A hyperbolic curve for a representative, typical experiment was fit using *K*_m_^fXII^ = 64 and [*fXIIa*]_max_ = 0.126 nM. The given *K*_m_ and [*fXIIa*]_m_ values are mean values (±SD) calculated using data from three independent experiments. **(D)** The effect of different platelet stimulation methods on platelet-dependent fXIIa formation in 20% plasma. The asterisks symbols (*) correspond to p<0.05. Table 1 represents the p values for comparing fXII activating capacities between activated and non-activated platelets.

To estimate platelet participation in fXII binding and activation, we used two experimental designs: purified fXII to exclude the contributions of other plasma enzymes as well as cofactors and fXII in plasma to better approximate physiological conditions. The activated platelets were predominantly in the PS-positive subpopulation (Fig. 1A) and bound purified fXII much better than non-activated platelets (S4A Fig.) in a calcium-independent manner (S4B Fig.) and in the 0 to 1 μM fXII concentration range (S4C Fig.). FXII binding by platelets under physiological conditions was 630±90 molecules/platelet (at 450 nM fXII). The values generated might include fXIIa binding because fXII may be partially activated during the assay.

In this and subsequent experiments, we converted platelet-related S2302 cleavage slopes (determined as shown in S3A Fig.) into fXIIa concentrations using a correlation coefficient obtained using purified fXIIa (S3C Fig.). The purified fXII results indicate that the platelet fXII-activating capacity is independent of other plasma factors. We did not observe a signal for activated platelets without fXII (Fig. 1B); hence, platelet secretion does not contain additional S2302-cleaving enzymes. Fig. 1C illustrates the dose-dependence of platelet-related fXIIa formation on the concentration of added fXII and shows that the apparent *K*_m_^fXII^ is approximately 50 nM, which is several-fold lower than the physiological fXII concentration and essentially lower than the approximately 300 nM *K*_m_ required for fXII auto-activation (S3D Fig.).

The results for fXII in plasma were qualitatively similar to the purified fXII data, except that the fXIIa yield was several-fold greater in plasma. Fig. 1D (platelet-independent fXII auto-activation was deducted) depicts the effect of different platelet stimulation on the platelet fXII-activating capacity. Platelets stimulated with a strong agonist (agonists that can induce PS exposure [21], [24], [28], see Table 1) acquired a reliable fXII-activating capacity, whereas non-activated or 2-MeS-ADP-stimulated platelets did not support fXII activation. The results in S3E Fig. confirm that the platelet activators (finally diluted 100-fold) did not participate in fXII activation in the absence of platelets. FXIIa inhibitor CTI sensitivity (Fig. 1D) was used as an additional control to distinguish fXIIa from other possible S2302 converters. Hence, the observed S2302 cleavage was not due to kallikrein or fXIa activity.

**Table 1:** Stimulation and platelet-dependent fXII activation.

Type of platelet activation	n	Total PS+ platelets (%)^a^	Factor XII activation		
			(fXIIa (nM))^a^	Statistical significance (p value)	
				*t*-test^c^	U-test^d^
Non-activated^b^	10	1.66±0.37	0.005±0.021		
Thrombin	7	15.3±6.3	1.57±1.17	0.0088	≤0.01
CRP	5	21.3±11.3	0.815±0.31	0.0065	≤0.01
Thrombin+CRP	3	43.8±7.99	0.739±0.28	0.0080	≤0.01
A23187	8	90.6±5.8	1.2±0.31	0.0006	≤0.01
MeS-ADP	3	2.22±0.65	0.075±0.014	0.396^nd^	>0.05^nd^

^a^ The data are the mean values (±SD) calculated for the corresponding number of independent experiments.

^b^ Platelets incubated in the presence of 2.5 mM CaCl_2_ were used as a negative control for activated platelets.

^c^ The results from Welch’s unpaired t-test to compare fXII-activation by activated platelets and non-activated platelets (the two-tailed p values are given).

^d^ The results from the Mann-Whitney U-test to compare fXII-activation by activated platelets and non-activated platelets (the one-tailed p values are given).

^nd^ Not significantly different results.

### The role of the platelet surface in activating fXII and contact coagulation

**Fig 2:**
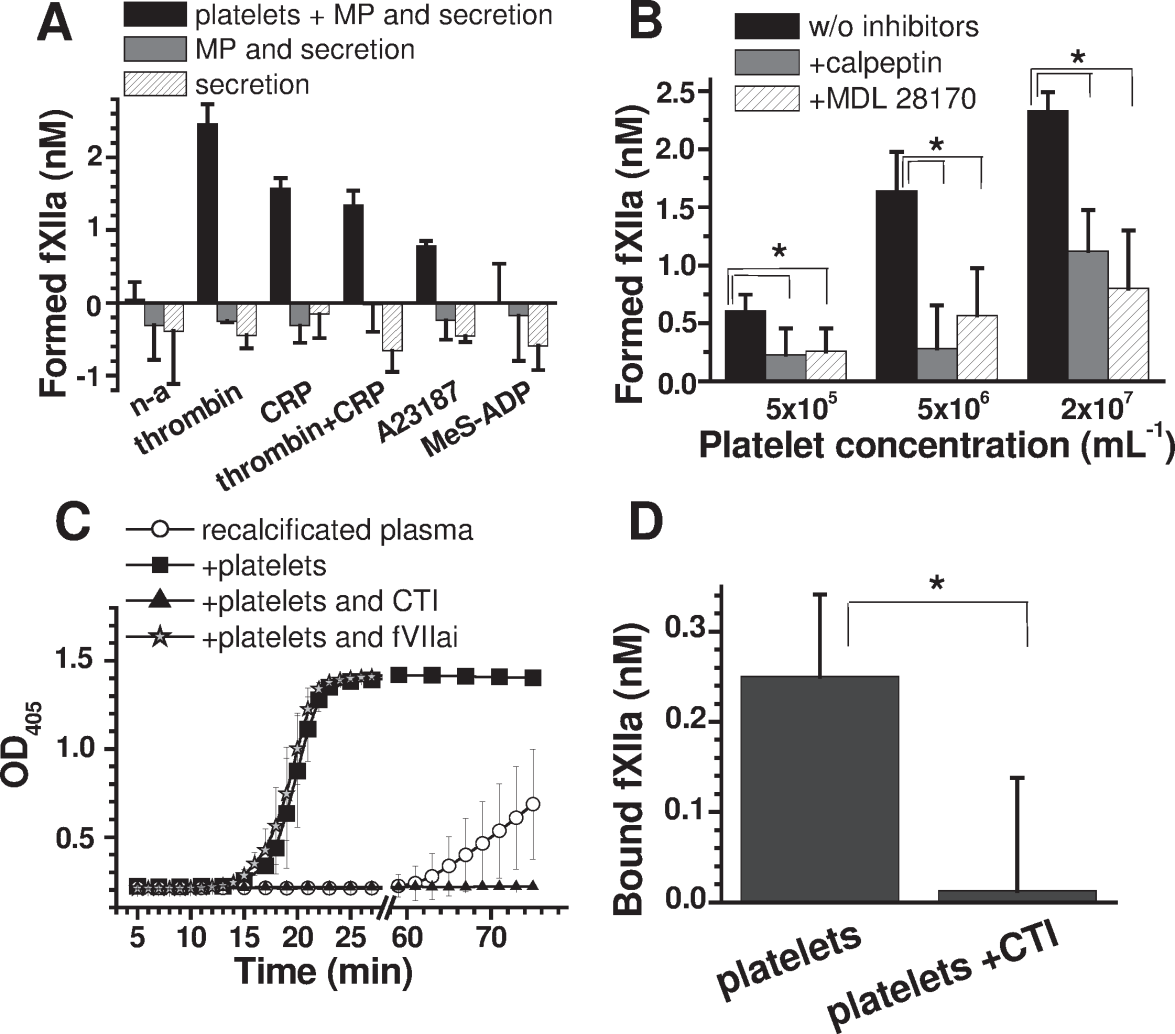
The roles of the platelet surface, MPs, and secretion in fXII activation and contact coagulation. **(A)** The effect of platelets and/or MP removal on fXIIa formation in 20% plasma (n = 3). After activation, the platelets were diluted to 4×10^6^/mL and centrifuged at 300 g for platelet precipitation and again at 16,000 g for MP precipitation [25]. **(B)** The effects of 200 μM calpeptin and MDL 28170 on fXIIa formation (in 20% plasma) by A23187-activated platelets after platelet secretion removal. **(C)** Clotting caused by A23187-activated, secretion-depleted platelets in recalcified plasma (n = 3). A23187-activated, secretion-depleted platelets (100 μL, 6×10^7^/mL) were supplemented with 500 μL of 90% recalcified plasma (see “Methods”), and clotting was measured as an increase in optical density at 405 nm; 50 nM fVIIai was used, and CTI was used at 200 μg/mL. **(D)** Retention of fXIIa by A23187-activated secretion-depleted platelets (n = 3). The platelets were activated with A23187 for 30 min and washed free of platelet secretion (see “Methods”). Next, 2×10^8^/mL platelets were incubated with 20% chelated plasma for 1 h before washing again to separate the platelets from the plasma and from soluble fXIIa. The activity of bound fXIIa was estimated using 200 μM S2302 for platelets at 2×10^7^/mL.

To evaluate the role of the platelet surface in fXII activation, we separated platelets from the MPs and platelet secretion through centrifugation [25]. The cellular component comprised most of the fXII-activating capacity (Fig. 2A); a 70–90% decrease in the platelet concentration (S5A and B Fig.) completely abolished fXIIa generation. Unexpectedly, the supernatant collected after the MPs were removed inhibited fXIIa auto-activation. However, through inhibiting MP formation using calpeptin and MDL 28170, which did not affect the percentage of PS-positive platelets (S5C and D Fig.), fXIIa generation by washed platelets was somewhat inhibited (Fig. 2B). The level of fXII binding to MP was comparable to the level of fXII binding to PS-positive platelets (S4D Fig.). These results implicate that platelet-released MPs participate in fXII activation.

To evaluate whether fXII activation by potently stimulated platelets is physiologically relevant for triggering coagulation, we performed experiments using MP-depleted, recalcified plasma supplemented with A23187-activated, washed platelets. The results in Fig. 2C demonstrate that platelets (1×10^7^/mL) efficiently trigger plasma clotting, which is entirely abrogated by the fXIIa inhibitor CTI and is insensitive to the TF antagonist fVIIai. Thus, activated platelets are likely a physiologically significant contact coagulation activator even without platelet-secreted material.

To determine whether activated fXII remains associated with the platelet surface, we incubated A23187-activated platelets with plasma, washed the platelets and then analysed the platelets for fXIIa activity. Fig. 2D indicates that the platelets retain approximately 1×10^4^ molecules of fXIIa/platelet, which is the same order of magnitude as the maximum fXIIa yield from a purified system (approximately 4.2×10^4^ molecules of fXIIa/platelet).

### FXII activation by platelets might be described by an auto-inhibitory mechanism

**Fig 3:**
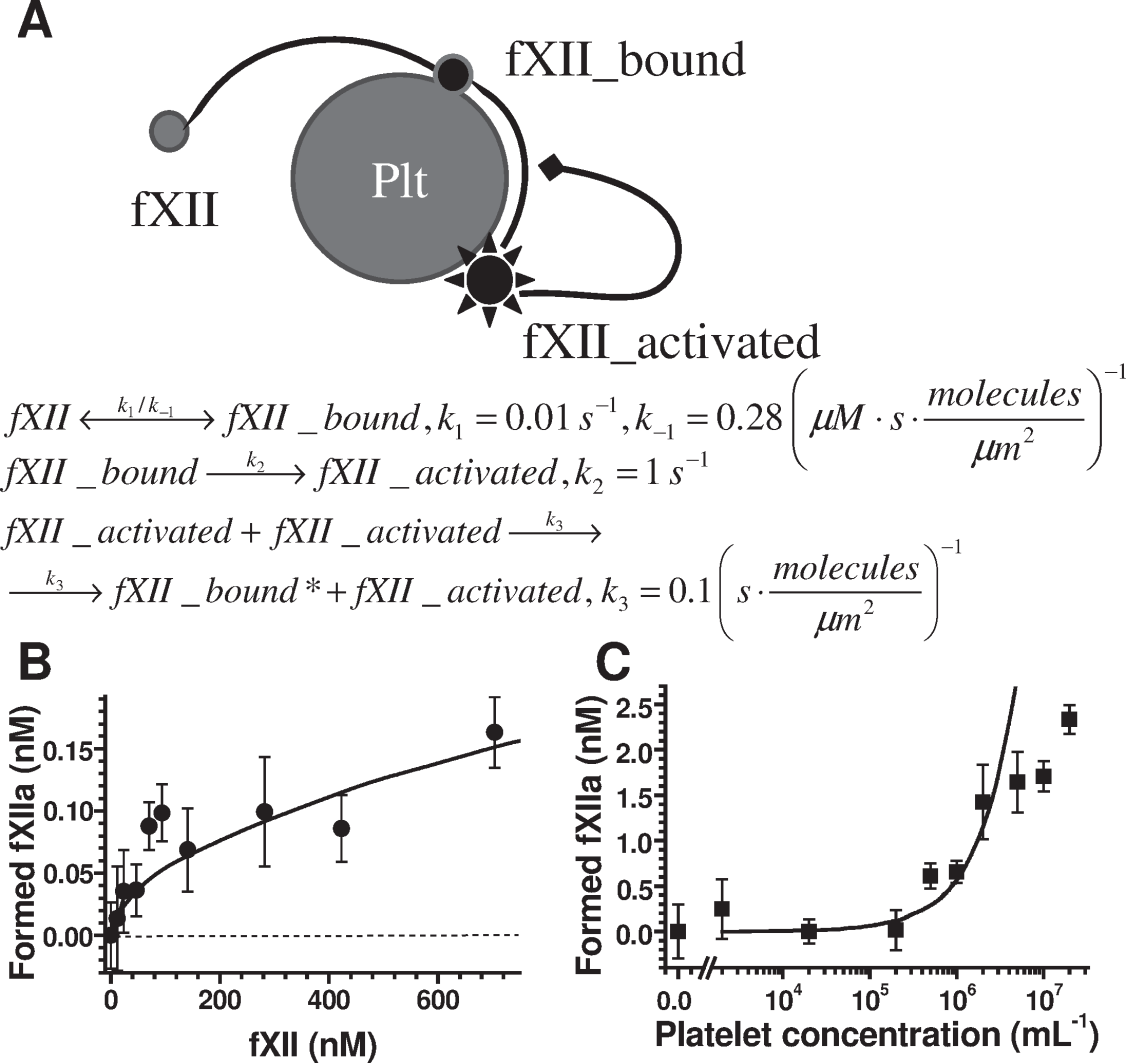
FXII activation model. **(A)** Computational model schematic for fXII activation on the platelet surface. Soluble fXII first binds the surface and is then activated; fXIIa can inhibit further activation through an auto-inhibition mechanism [26]. The reaction rate constants (*k*_i_) for the model were estimated based on the data in Fig. 1C and S4C Fig., and the surface area of an activated platelet was considered 50 μm^2^. **(B)** Platelet-dependent fXIIa formation in buffer as a function of the fXII concentration added. The black circles correspond to the experimental data (±SD for three independent experiments, n = 3); the curve corresponds to the computational model. The consistency between the data and model curve confirms the fXII auto-inhibition hypothesis. **(C)** The dose-dependence of fXIIa formation in 20% plasma for A23187-activated platelets after platelet secretion removal. The platelets were activated at 2×10^8^/mL and diluted to the indicated concentrations for further reaction with plasma. The black squares represent the experimental data (n = 3), and the curve represents the computational model. The consistency between the data and model curve confirms the reliability of the model.

The experimental data above shows several unusual features of fXII activation by PS-positive platelets. First, fXII binding was highly unsaturated at a physiological concentration (S4C Fig.); however, fXII activation was saturated at 100 nM (Fig. 1C). Second, the maximum concentration of fXIIa formed (approximately 0.14 nM for 2×10^6^/mL platelets, Fig. 1C) corresponds to approximately 4.2×10^4^ fXIIa molecules/platelet, which is approximately two orders of magnitude greater than the value generated from binding experiments (∼6.3×10^2^ molecules/platelet). To explain these discrepancies, we further investigated this phenomenon using a computer model (Fig. 3). FXII activation on hydrophilic surfaces involves an auto-inhibition mechanism [26], which we considered when interpreting our results (S4C Fig. and Fig. 1C), and we constructed a computer model for fXII binding using a platelet surface and additional activation through an auto-inhibition mechanism (Fig. 3A).

This model allowed us to quantitatively describe the experimental data (Fig. 3B); thus, both the binding and activation data were united, and the above-described discrepancies were described. The model was also used to predict the exponential dependence of fXII activation on platelet concentration (Fig. 3C, smooth curve). To test this notion, we examined the dose-dependence of fXIIa formation using A23187-activated platelets, which were washed free of platelet secretion (Fig. 3C); the experimental data were consistent with a calculated curve for the platelet concentration range 0–5×10^6^/mL. These data confirmed fXII activation by the platelet surface through an auto-inhibition mechanism, which is similar to fXII activation by the hydrophilic surface [26].

### The roles of PS, poly-P, and α-granule secretion in surface-associated fXII activation

**Fig 4:**
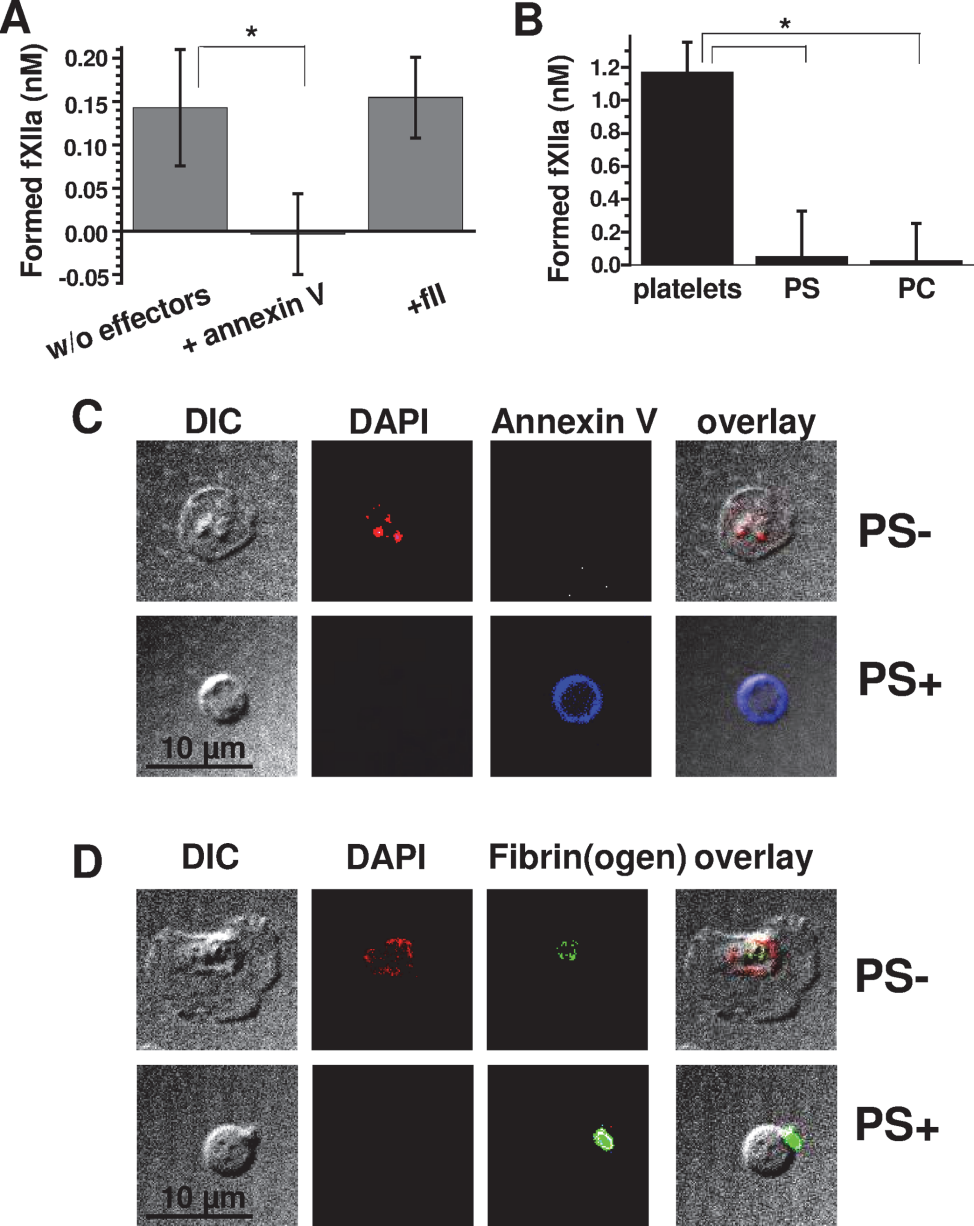
The role of PS and poly-P in surface-associated fXII activation by platelets. **(A)** The effect of 20 μg/mL annexin V or 1.5 μM prothrombin on 280 nM fXII activation (in the presence of 2.5 mM CaCl_2_) by A23187-activated (secretion-depleted) platelets (n = 3). **(B)** Comparison of the fXIIa-generating capacities for A23187-activated platelets (at 2×10^6^/mL) and 4 μM PS- or PC-liposomes (depicted as PS and PC, respectively) in 20% chelated plasma. The mean values (±SD) were calculated from three independent experiments (n = 3). **(C)** Confocal microscopy analysis of poly-P localisation in activated platelets with 2.5 mM calcium. DAPI was used as poly-P marker [27], and AlexaFluor-647-annexin V was used as a marker for procoagulant PS-positive platelets (labelled “PS+”). PS-negative activated platelets are labelled “PS-” (n = 3). **(D)** Confocal microscopy analysis of poly-P localisation in activated platelets without 2.5 mM calcium. FITC-anti-fibrin(ogen) antibody was used as an activated platelet marker (n = 3). The images in (C) and (D) were collected at an ∼2 μm z-depth over the fibrinogen surface to reduce non-specific fibrinogen immunofluorescence.

To determine whether PS contributes to platelet-dependent fXIIa generation or whether it is merely concurrent with manifestation of an additional necessary factor, we examined the effect of annexin V on purified fXII activation. The results generated using A23187-activated platelets after washing (Fig. 4A) demonstrate that annexin V fully abrogates platelet-dependent fXII activation. Interestingly, at its physiological concentration, prothrombin (fII) did not inhibit fXII activation (Fig. 4A), which suggests preferential fXII binding and that the interaction between fXII and activated platelets may be physiologically relevant. Furthermore, synthetic microvesicles containing 20% PS did not activate fXII under these conditions (Fig. 4B), which is consistent with earlier investigations [29], [30]; this result implies that an additional factor may co-express with PS during fXII-activation.

To determine whether poly-P remain associated with the platelet surface, we used confocal microscopy images of non-activated (S6A and B Fig.) and activated PS-negative or PS-positive platelets (Fig. 4C and D) treated with DAPI, which was previously optimised for studying poly-P localisation [27] and specifically examined under the conditions herein (S6C Fig.). The results confirm DAPI localisation inside the dense granules of PS-negative and non-activated platelets. In contrast, poly-P retention was not observed for the PS-positive platelet surface, regardless of whether calcium was added.

**Fig 5:**
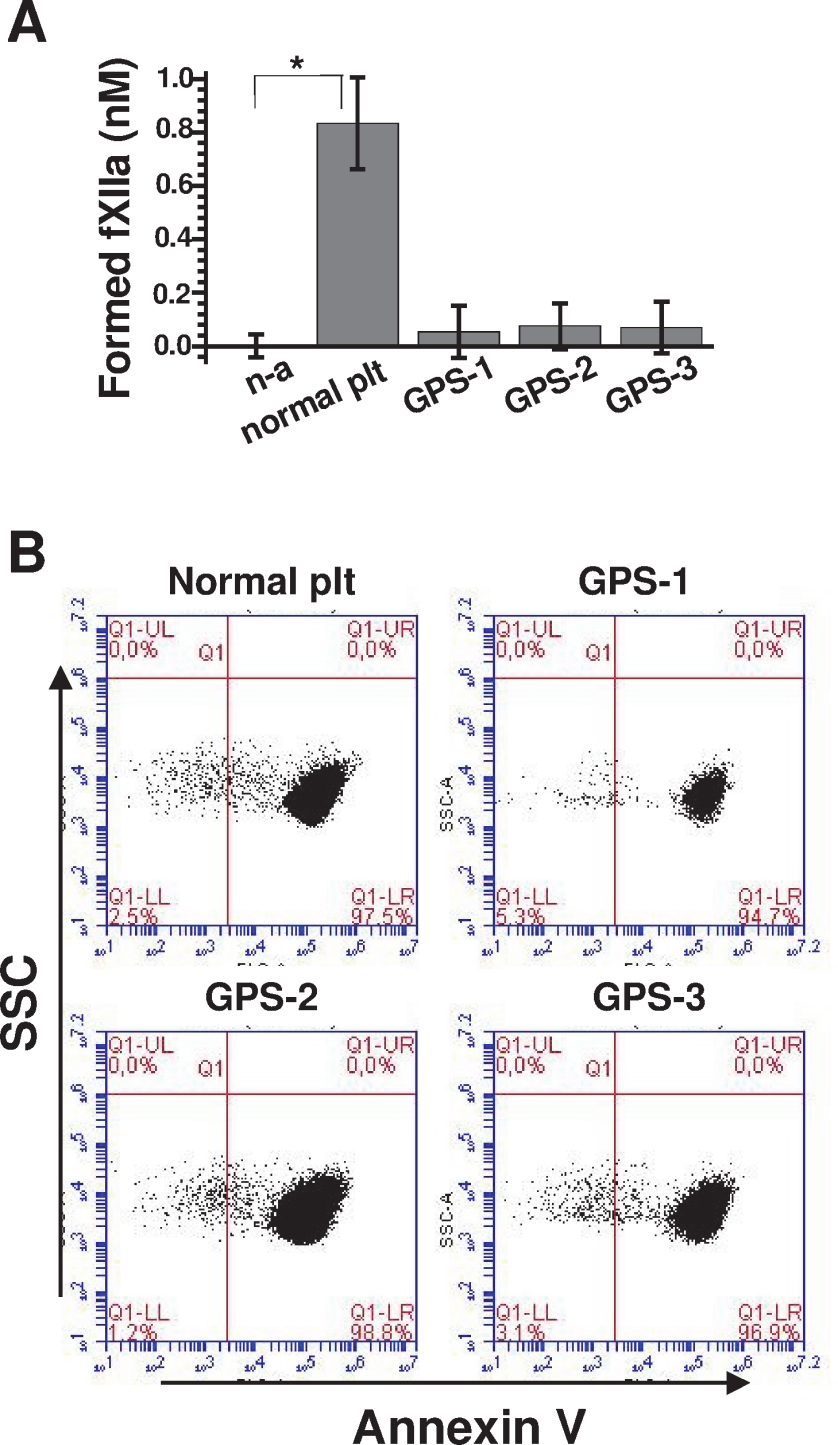
Comparison of fXII activation by platelets from a normal donor and GPS patients. **(A)** Comparison of the fXIIa-generating capacities for A23187-activated platelets (at 2×10^7^/mL) from a normal donor (labelled “normal plt”) and three grey platelet syndrome patients (labelled GPS-1, GPS-2, and GPS-3, respectively). **(B)** Flow-cytometry dot plots demonstrating the significant PS exposure in the analysed platelet preparations upon activation with A23187.

Many α-granule proteins remain associated with the PS-positive platelet surface through a specific’cap' structure [31]. To elucidate a potential role for α-granule secretion in platelet fXII-activation, we evaluated fXII activation using A23187-activated, washed ‘grey’ platelets from three GPS patients. Fig. 5A shows that fXII was not activated in the ‘grey’ platelet preparations that contained >90% of PS-positive platelets (Fig. 5B). This result suggests that α-granule secretion might contain the factor that supports fXII activation.

### Platelet secretion inhibits fXIIa

**Fig 6:**
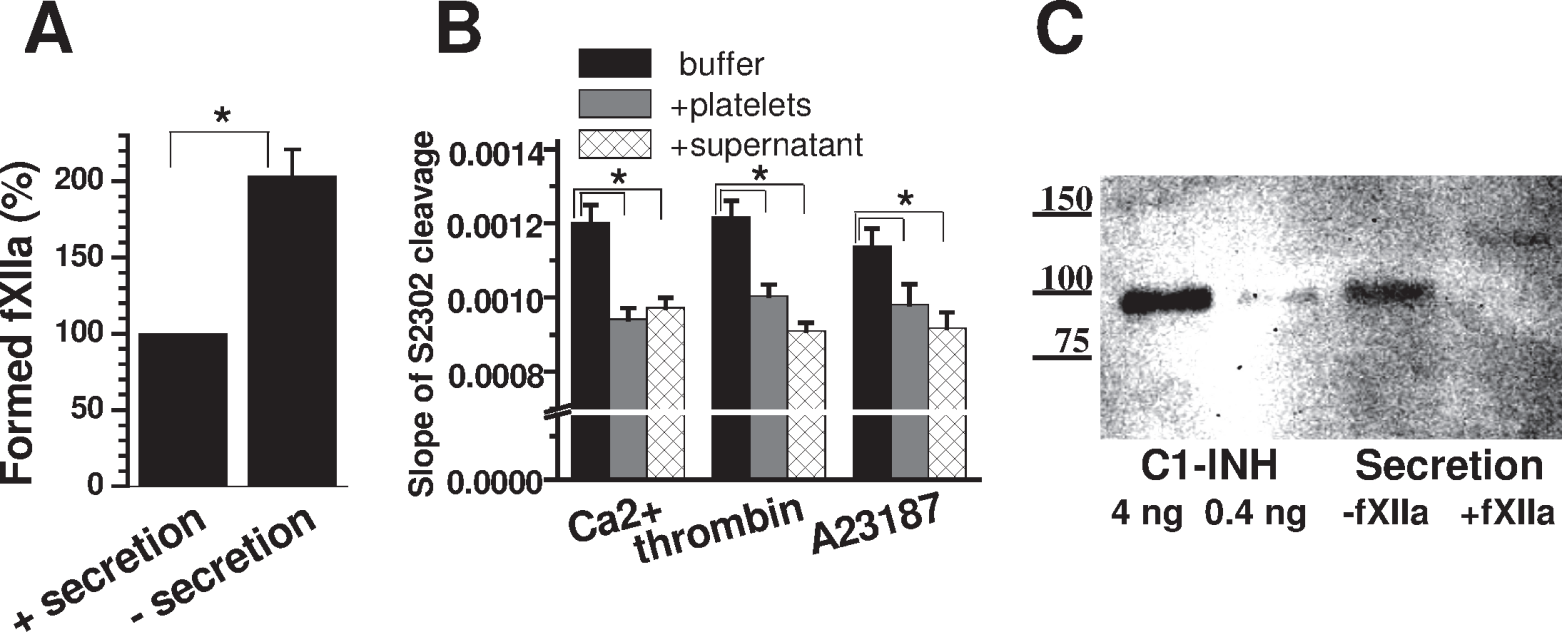
Inhibition of fXIIa activity through platelet secretion. **(A)** The effect of platelet secretion removal on platelet-related fXII activation (means±SD for three independent experiments, n = 3). A portion of the A23187-activated platelet suspension was diluted to 4×10^6^/mL and mixed 1:1 with 40% chelated plasma to measure the initial fXIIa-generating capacity in the presence of platelet secretion (which was estimated at 100%). The remaining platelets were washed free from platelet secretion, diluted, and analysed. **(B)** The effect of platelet secretion on 2 nM fXIIa activity (n = 3). The reaction in buffer A without platelets was compared with the reaction in the presence of 2×10^6^/mL activated platelets or platelet secretion (at an equivalent dilution). **(C)** Western blot analysis of purified C1-INH (labelled “C1-INH”) and platelet secretion (labelled “Secretion”). Supernatant containing platelet secretion was incubated with 25 nM fXIIa, and the total protein was concentrated 20-fold. After resolution using 4–10% SDS-PAGE gels and subsequent Western blotting, the bands were developed using the ECL method. The figures on the left depict the molecular masses of markers measured in kDa (n = 4).

As revealed above, platelet material secreted into a liquid phase under the conditions herein inhibits fXIIa generation (Fig. 2A). To elucidate the level of inhibition, we directly compared fXIIa generation from A23187-stimulated platelets with or without the secretion. Fig. 6A implies that secretion causes at least a two-fold inhibition; Fig. 6B further indicates that this inhibition is directed at the activated factor, and the inhibitor may be released by non-activated platelets. These circumstances suggest that this inhibition may be due to the C1-inhibitor (C1-INH), which is involved in pro-inflammatory platelet-related reactions and can be released independent of haemostatic platelet activation [32], [33]. Western blotting (Fig. 6C) confirmed the presence of C1-INH under the conditions herein and that C1-INH was covalently bound to a protein with a molecular weight similar to the fXIIa light-chain fragment. More importantly, under the conditions herein, platelets activated in the presence of fXII generate complexes between C1-INH and fXIIa fragments, which were demonstrated through immuno-precipitation and subsequent Western blotting studies (S7 Fig.). Therefore, the observed inhibition may be at least partially due to released C1-INH. However, the presence of another inhibitor(s) cannot be excluded based on these results.

### Platelet-secreted inhibitors washed through blood flow can regulate contact activation

**Fig 7:**
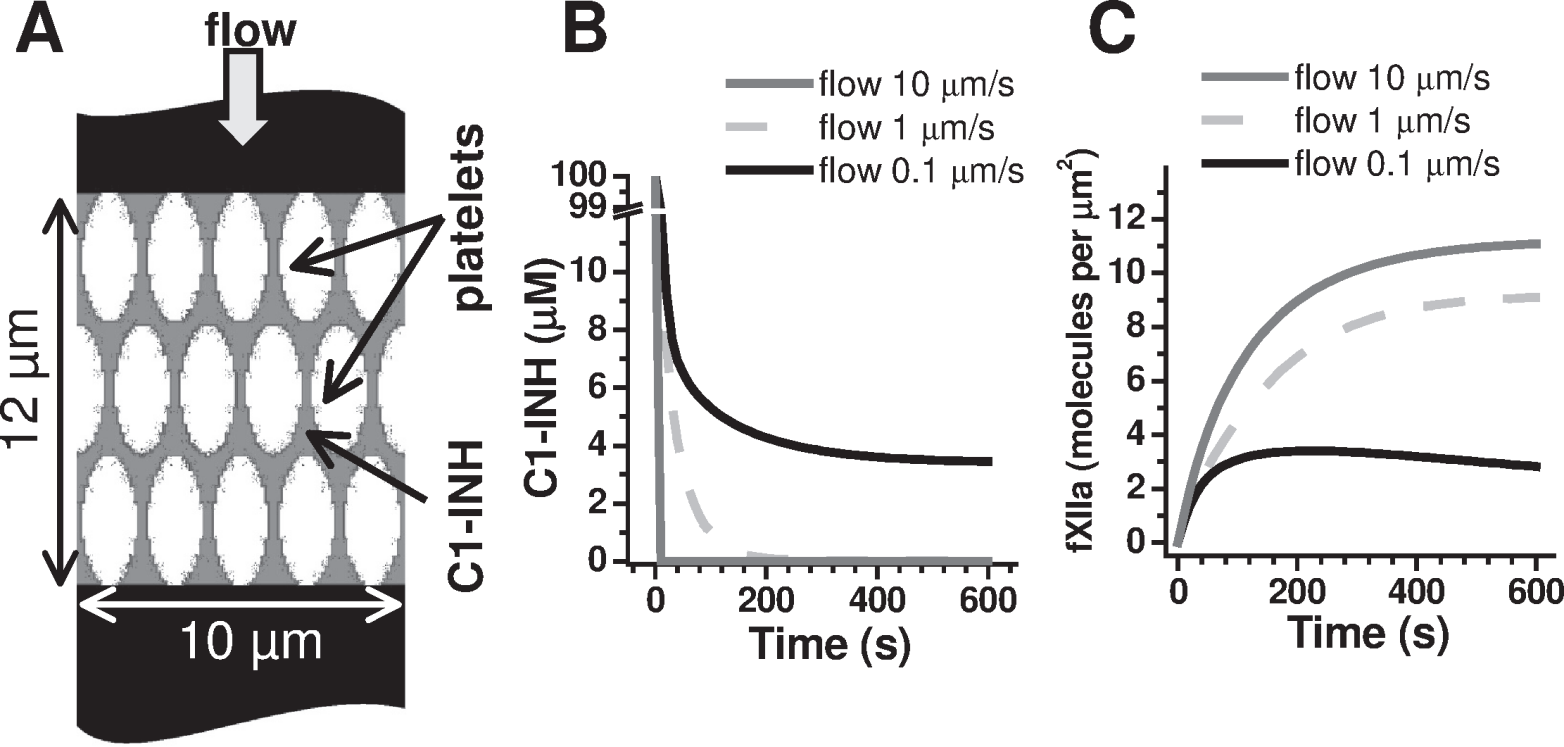
Computer simulation of C1-inhibitor washout. (**A**) We used a scheme that depicts the platelet aggregate to test the C1-INH washout hypothesis. The platelet concentration inside the thrombi was considered 1 per 15 fl. The initial C1-inhibitor distribution in the aggregate for the simulations is shown in grey, and at values ranging from 0.1 to 10 μm/s, the flow penetrated the aggregate. At t = 0, C1-INH at 100 μM (estimated from ref. [33]) and fXII at 450 nM appeared simultaneously. Factor XII could be activated on the platelet surface, and C1-INH could diffuse through the aggregate (D = 10 μm^2^/s, based on the molecular weight) and move due to the flow. The fXIIa diffusion was assumed negligible because fXII activation is surface-associated ([10] and this study), and typically, fXIIa is tightly bound to the activation surface [39]. The C1-INH action was described using a mass action equation with the reaction constant [34] 0.00366 μM^-1^s^-1^. (**B**) Time-course of the distance-averaged [C1-INH] for various flow velocities. (**C**) Time-course of the surface-averaged [fXIIa] for various flow velocities. The C1-INH and fXIIa spatial distributions were governed by a set of differential equations, which were solved using the finite volume solver available within the Virtual Cell environment.

Based on the above-described combination of surface-associated fXII activation and fXIIa inhibition through secretion, we propose that the C1-INH efficiency in thrombi might depend on the rate of blood flow, which may washout C1-INH. Although physiological C1-INH plasma concentrations reach approximately 2 μM [34], the local C1-INH concentration in thrombi may be much greater. To test our suggestion, we developed a mathematical model for C1-INH washout (Fig. 7A). We considered each platelet capable of fXII activation with the above-described kinetics (Fig. 3A). Assuming the observed increased platelet concentration inside thrombi (>7×10^8^/mL, Fig. 7A) and that one platelet can release 0.2 fg of C1-INH (31), the local C1-INH concentration can transiently reach approximately 100 μM. The computer simulation results (Fig. 7B and C) demonstrate that, under low blood flow (0.1 μm/s), the released C1-INH dissipates due to diffusion in the intracellular space until the concentration reaches approximately 5 μM within approximately 2 min (Fig. 7B, black curve). Hence, the aggregate retains approximately 7 μM C1-INH (including plasma C1-INH), which is at several-fold excess of its plasma levels; this level of C1-INH might increase the inactivation rate for the fXIIa formed.

Furthermore, a 100-fold-increased flow leads to 99% washout of the secreted C1-INH within 20 s (Fig. 7B and C, grey curves). This level of plasma C1-INH is insufficient for rapid fXIIa inhibition due to low-inactivation at the pseudo-first-order rate constant 37.410^-2^ min^-1^ [34]; thus, it might be critical for triggering coagulation by fXIIa formed in thrombi. These results support the hypothesis proposed and imply that platelet-surface-dependent fXII activation and secretion-dependent inactivation might combine to function as a flow-sensitive regulator.

## Discussion

Although several studies have examined the participation of activated platelets or platelet-derived material in fXII activation [9], [10], [13], [11], [12], the overall role of platelets in fXII activation remains disputed [15], [17]. Our results demonstrate that the surface of PS-positive, but not PS-negative, platelets significantly activates fXII under physiological conditions and that the surface-associated fXII activator may originate in the alpha-granules. Unexpectedly, the overall effect of soluble platelet secretion on fXII activation was inhibitory, not activating. The combined two opposing effects might explain the discrepancy between earlier studies.

The role of platelet subpopulations in fXII binding and activation is considered here for the first time. Three lines of evidence suggest that PS exposure to the procoagulant subpopulation could be significant: i) PS-positive platelets bound fXII approximately 5-fold better than PS-negative platelets; ii) fXII activation was only exhibited upon phosphatidylserine exposure; and iii) annexin V, which is a specific marker and inhibitor of PS, almost fully inhibited platelet-dependent fXII activation. However, the inability of synthetic PS-vesicles to activate fXII implies that PS is insufficient for fXII activation. The ‘grey’ platelet results strongly suggest that α-granule secretion may play an essential role. Nevertheless, PS participates in fXIIa binding and retention [29], which may be important for supporting fXII activation, its interaction with other coagulation factors [21], [35], [36], and clotting localisation within platelet aggregates.

The precise mechanism by which the platelet surface may activate fXII is particularly interesting. Due to activation by foreign surfaces and significant potentiation by kallikrein [37], spontaneous, steady-state fXIIa concentrations in platelet-poor plasma can reach approximately 0.6 nM [38]. Thus, when analysing experiments involving only plasma, it is impossible to conclude whether the platelet surface directly activates fXII or simply facilitates activation through additional plasma components. Nevertheless, the results from purified fXII (Fig. 1), fXIIa retention by PS-exposing platelets (Fig. 2D), previous reports on tight binding between fXIIa and the activation surface [39], and the fXII activation dependence on binding site density (as predicted by the computational model) all favour direct fXII activation by the platelet surface.

An additional finding from the study on fXIIa-inhibition with regard to platelet secretion was unexpected because this inhibition was not considered in recent publications [10], [11], [12], [15]; however, this finding was not entirely surprising. Previous studies have demonstrated that platelets can release and retain the main fXIIa inhibitor C1-INH [32], [33]. However, our finding is significant because it suggests that an important aspect for the overall role of platelet secretion in fXIIa regulation should be reconsidered. In particular, our data suggest that the fXII-activating and fXIIa-inhibiting capacities may be segregated between the platelet surface and soluble platelet-released fraction, respectively. Combined, the two opposing reactions prompted us to consider whether the effect of fXIIa in thrombi depends on the rates of blood flow and C1-INH washout. Based on experimental data, our computer simulations confirm that, upon simultaneous fXII activation and secretion, the released C1-inhibitor may be rapidly washed out from physiological-size aggregates at relevant arterial thrombi filtration velocities. In particular, this finding might relate to arterioles with rapid flow, wherein XIIa prothrombotic action may increase due to rapid C1-INH removal from primary platelet aggregates. However, under low blood flow, the C1-INH concentration remains sufficiently high for rapid fXIIa inhibition, which may occur both in vessels with low blood flow and in haemostatic plugs in the absence of flow.

These considerations may explain why fXIIa is important for thrombosis but negligibly important for haemostasis.

## Supporting Information

S1 Fig FITC-fXII, CTI, and fVIIai preparation controls used in the study. **(A)** The effect of 200 mg/mL CTI on the rate of 200 M S2302 cleavage by fXIIa in buffer A. **(B)** The CTI addition timing and the level of its effect on platelet-associated fXIIa generation (S2302 cleavage). The platelets were activated with A23187 and applied to a reaction with 20% chelated plasma. We added CTI (200 mg/mL) to the reaction with platelets with simultaneously or 15 min thereafter (n = 3). **(C)** The effect of 50 nM fVIIai on 90% recalcified plasma clotting by 0.75 pM TF. **(D)** SDS PAGE analysis of the FITC-fXII preparation used in binding assays. The preparation was applied to 7.5% SDS PAGE, and the protein bands were developed using FITC fluorescence (left image, 4-sec exposure) and Coomassie R250 (right image). The figures on the left indicate the percent FITC fluorescence for the corresponding bands. The figures between two images represent molecular mass markers (kDa).
(TIF)

S2 Fig Flow-cytometry dot plots for non-activated and A23187-activated platelets; MP determination. MP formation was inhibited by 200 μM calpeptin.
(TIF)

S3 Fig Platelet-dependent fXIIa formation. **(A)** A typical experiment where the platelet contributes to the total fXII activation through reacting with 20% plasma (the platelets were activated by 10 nM thrombin). The curve with grey squares corresponds to platelet independent fXII auto-activation; white triangles—total fXII activation in the presence of platelets; asterisk—the optical density decrease due to platelet aggregation; black circles—calculated platelet-dependent fXII activation. **(B)** The effect of 200 mM S2302 on fXII activation. Factor XII activation was initiated by mixing A23187-activated platelets with plasma (through 2×106/mL and 20%, correspondingly). S2302 was added to the different mixture aliquots with the platelets simultaneously or 5 and 20 min thereafter. We initiated the fXIIa activity measurements simultaneously for all aliquots 20 min after the reaction began. The curves correspond to the total reaction, including both platelet-dependent and platelet-independent fXIIa activity (the change in OD405 in the absence of S2302 was subtracted). **(C)** The dose-dependence of the S2302 conversion rate (optical density increase at 405 nm) on the concentration of fXIIa added. The reaction was measured in buffer A with 200 mM S2302. Each point shows mean value (±SEM) for three experiments. The calibration curve generated was used to determine the correlation coefficient and convert the S2302 cleavage rate into the fXIIa concentration. **(D)** Determination of the platelet-dependent and platelet-independent fXIIa formation parameters upon reaction with purified fXII (n = 3). White circles correspond to platelet-independent fXII auto-activation; black circles correspond to the total reaction in the presence of platelets. The hyperbolic curves for the representative, typical experiment were fit using the given parameters. **(E)** The effect of 100-fold diluted platelet activators on platelet-independent S2302 cleavage in 20% plasma. We terminated 10 nM thrombin activation through adding 100 nM PPACK. Further, these concentrations were 100-fold lower upon platelets dilution for the reaction with plasma.
(TIF)

S4 Fig FXII binding to platelets and MPs. **(A)** A histogram of FITC-fXII (450 nM) binding to platelets: 1—control (platelets without fXII); 2—non-activated platelets; 3—activated platelets. **(B)** The effect of EDTA on 450 nM FITC-fXII binding to a non-activated or an activated platelet membrane. **(C)** Correlation between fXII binding and its added concentration for non-activated and activated PS-positive and PS-negative platelets (n = 3). **(D)** FITC-fXII (450 nM) binding to MPs compared with binding to PS-positive platelets. We identified the microparticles in flow-cytometry dot plots as shown in S2 Fig. The mean values (±SD) were calculated for three independent experiments (n = 3).
(TIF)

S5 Fig Platelets and MP removed; the effects of calpeptin and MDL 28170. The microparticles were identified in flow-cytometry dot plots as shown in S2 Fig. **(A)** The level of remaining platelets after centrifugation. **(B)** The level of remaining MPs after centrifugation. In panels (A and B), the mean values (±SD) were calculated for three independent experiments (n = 3). **(C)**. The effects of calpeptin and MDL 28170 on the variation in the MP to platelets ratio (n = 3). **(D)** The inhibitors’ effects on PS-positive platelet formation (n = 3).
(TIF)

S6 Fig Confocal microscopy analyses of poly-P localisation in non-activated platelets. **(A)** Co-localisation of DAPI with mepacrine confirms dense granule staining. **(B)** Different localisation for DAPI and TMRM confirms that, under the conditions herein, DAPI does not stain mitochondria (or mitochondrial DNA) (n = 3). **(C)** Fluorescence spectra for 50 mM DAPI and DAPI-poly-P complex under the conditions used herein (buffer A, excitation at 405 nm). The poly-P preparation with the chain length 1–200 was kindly provided by Dr. Kulakovskaya T.V. (G.K. Skryabin Institute of Biochemistry and Physiology of Microorganisms, RAS, Pushchino, Russia).
(TIF)

S7 Fig SDS-PAGE and Western blot analyses of C1-INH and its complexes with fXIIa fragments obtained through immuno-precipitation from a platelet suspension, which was activated in the presence or absence of added fXII. **(A)** SDS PAGE analysis (silver staining); **(B)** Western blot analysis using antihuman C1-INH antibody as the primary antibody; **(C, D)** Western blot analysis using antihuman factor XII antibody as the primary antibody. Lanes *1* and *2* correspond to purified C1-INH preparation (5 and 50 ng, respectively); lanes *3*—molecular mass marker (the figures on the left depict the molecular masses in kDa); lanes *4* and *5*—immuno-precipitation results obtained in the absence or presence of added fXII, respectively; lane *6*—the control purified fXII immuno-precipitation performed in the absence of platelets. The arrows indicate the covalent complexes formed between C1-INH and fXII fragments under conditions herein.
(TIF)
