# Supplementary figures and images for: Platelet Surface-Associated Activation and Secretion-Mediated Inhibition of Coagulation Factor XII
Source: PLoS One. 2015 Feb 17;10(2):e0116665. doi: 10.1371/journal.pone.0116665 (PMC4331558; doi:10.1371/journal.pone.0116665)

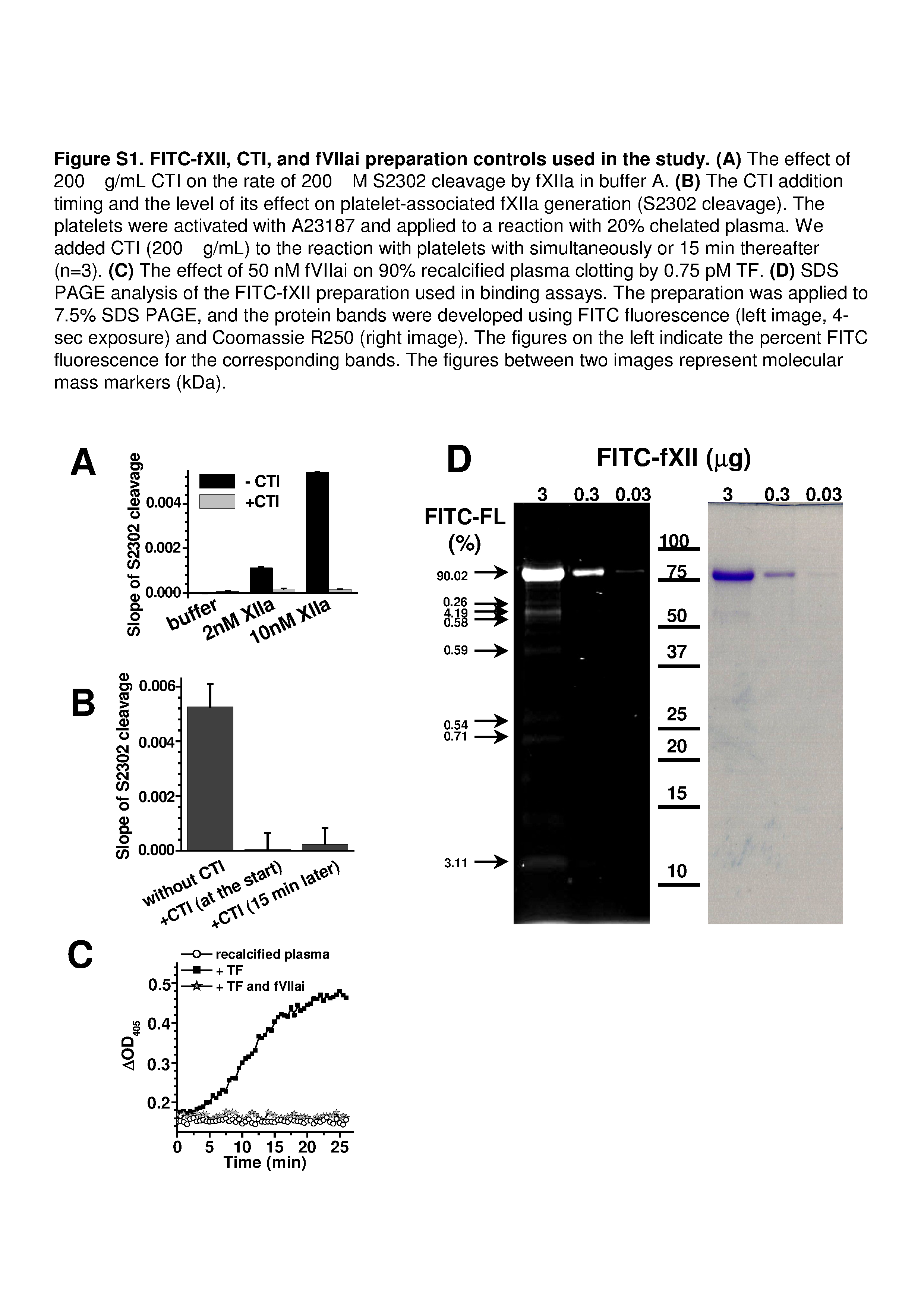

Supplement: S1 Fig — (A) The effect of 200 mg/mL CTI on the rate of 200 M S2302 cleavage by fXIIa in buffer A. (B) The CTI addition timing and the level of its effect on platelet-associated fXIIa generation (S2302 cleavage). The platelets were activated with A23187 and applied to a reaction with 20% chelated plasma. We added CTI (200 mg/mL) to the reaction with platelets with simultaneously or 15 min thereafter (n = 3). (C) The effect of 50 nM fVIIai on 90% recalcified plasma clotting by 0.75 pM TF. (D) SDS PAGE analysis of the FITC-fXII preparation used in binding assays. The preparation was applied to 7.5% SDS PAGE, and the protein bands were developed using FITC fluorescence (left image, 4-sec exposure) and Coomassie R250 (right image). The figures on the left indicate the percent FITC fluorescence for the corresponding bands. The figures between two images represent molecular mass markers (kDa). (TIF) [file pone.0116665.s001.tif]

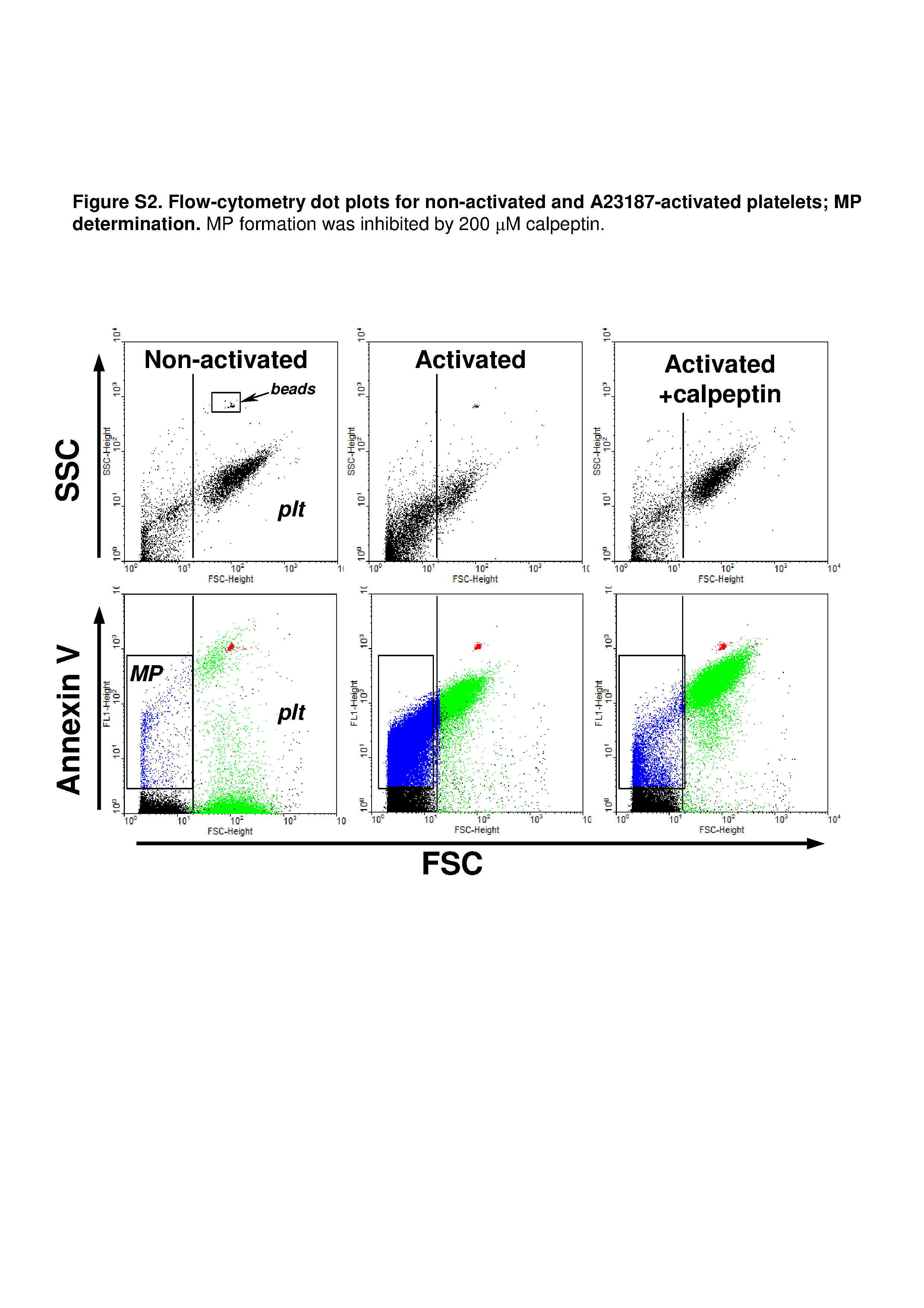

Supplement: S2 Fig — MP formation was inhibited by 200 μM calpeptin. (TIF) [file pone.0116665.s002.tif]

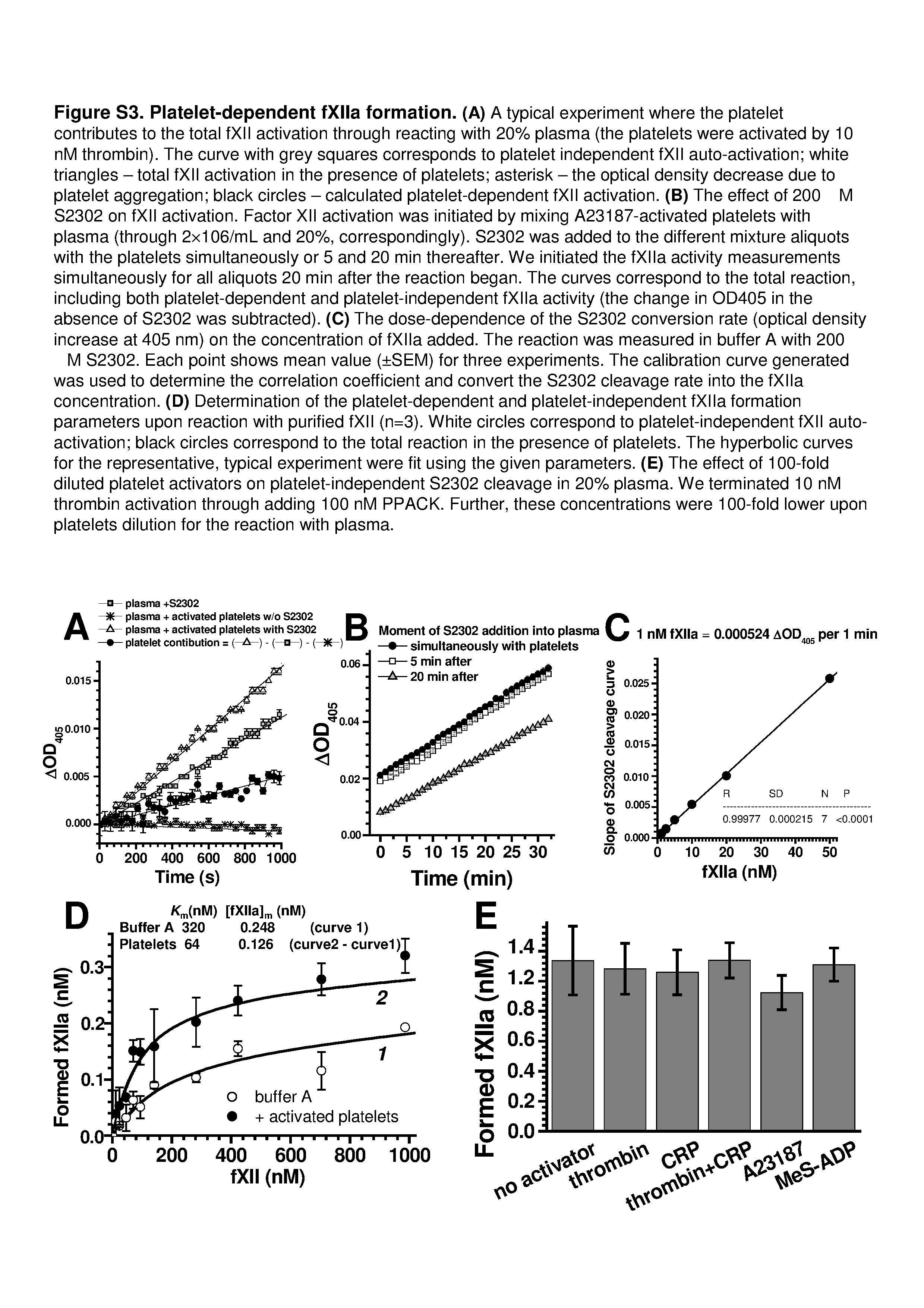

Supplement: S3 Fig — (A) A typical experiment where the platelet contributes to the total fXII activation through reacting with 20% plasma (the platelets were activated by 10 nM thrombin). The curve with grey squares corresponds to platelet independent fXII auto-activation; white triangles—total fXII activation in the presence of platelets; asterisk—the optical density decrease due to platelet aggregation; black circles—calculated platelet-dependent fXII activation. (B) The effect of 200 mM S2302 on fXII activation. Factor XII activation was initiated by mixing A23187-activated platelets with plasma (through 2×10^6^/mL and 20%, correspondingly). S2302 was added to the different mixture aliquots with the platelets simultaneously or 5 and 20 min thereafter. We initiated the fXIIa activity measurements simultaneously for all aliquots 20 min after the reaction began. The curves correspond to the total reaction, including both platelet-dependent and platelet-independent fXIIa activity (the change in OD405 in the absence of S2302 was subtracted). (C) The dose-dependence of the S2302 conversion rate (optical density increase at 405 nm) on the concentration of fXIIa added. The reaction was measured in buffer A with 200 mM S2302. Each point shows mean value (±SEM) for three experiments. The calibration curve generated was used to determine the correlation coefficient and convert the S2302 cleavage rate into the fXIIa concentration. (D) Determination of the platelet-dependent and platelet-independent fXIIa formation parameters upon reaction with purified fXII (n = 3). White circles correspond to platelet-independent fXII auto-activation; black circles correspond to the total reaction in the presence of platelets. The hyperbolic curves for the representative, typical experiment were fit using the given parameters. (E) The effect of 100-fold diluted platelet activators on platelet-independent S2302 cleavage in 20% plasma. We terminated 10 nM thrombin activation through adding 100 nM PPACK. Further, t [file pone.0116665.s003.tif]

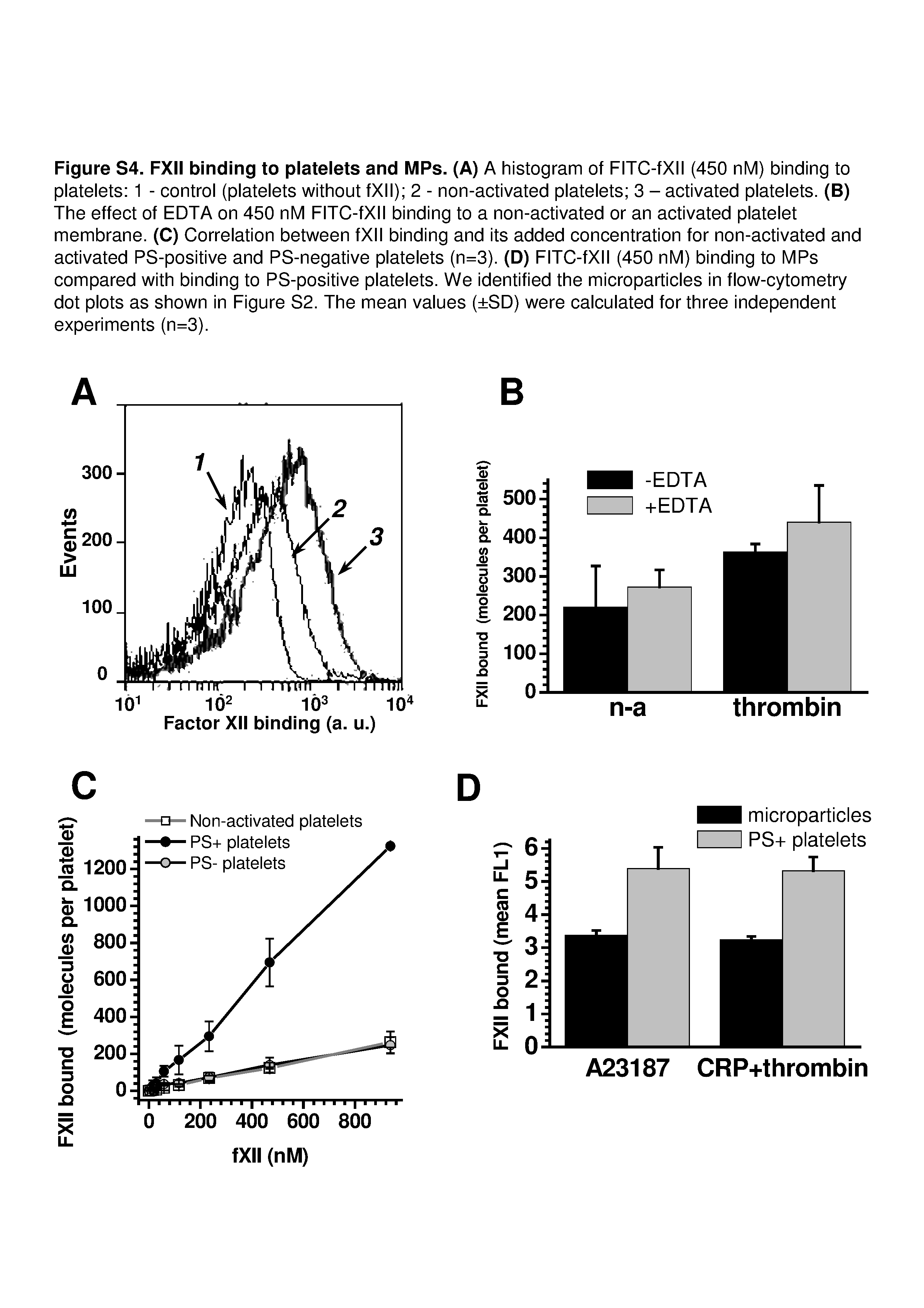

Supplement: S4 Fig — (A) A histogram of FITC-fXII (450 nM) binding to platelets: 1—control (platelets without fXII); 2—non-activated platelets; 3—activated platelets. (B) The effect of EDTA on 450 nM FITC-fXII binding to a non-activated or an activated platelet membrane. (C) Correlation between fXII binding and its added concentration for non-activated and activated PS-positive and PS-negative platelets (n = 3). (D) FITC-fXII (450 nM) binding to MPs compared with binding to PS-positive platelets. We identified the microparticles in flow-cytometry dot plots as shown in S2 Fig. The mean values (±SD) were calculated for three independent experiments (n = 3). (TIF) [file pone.0116665.s004.tif]

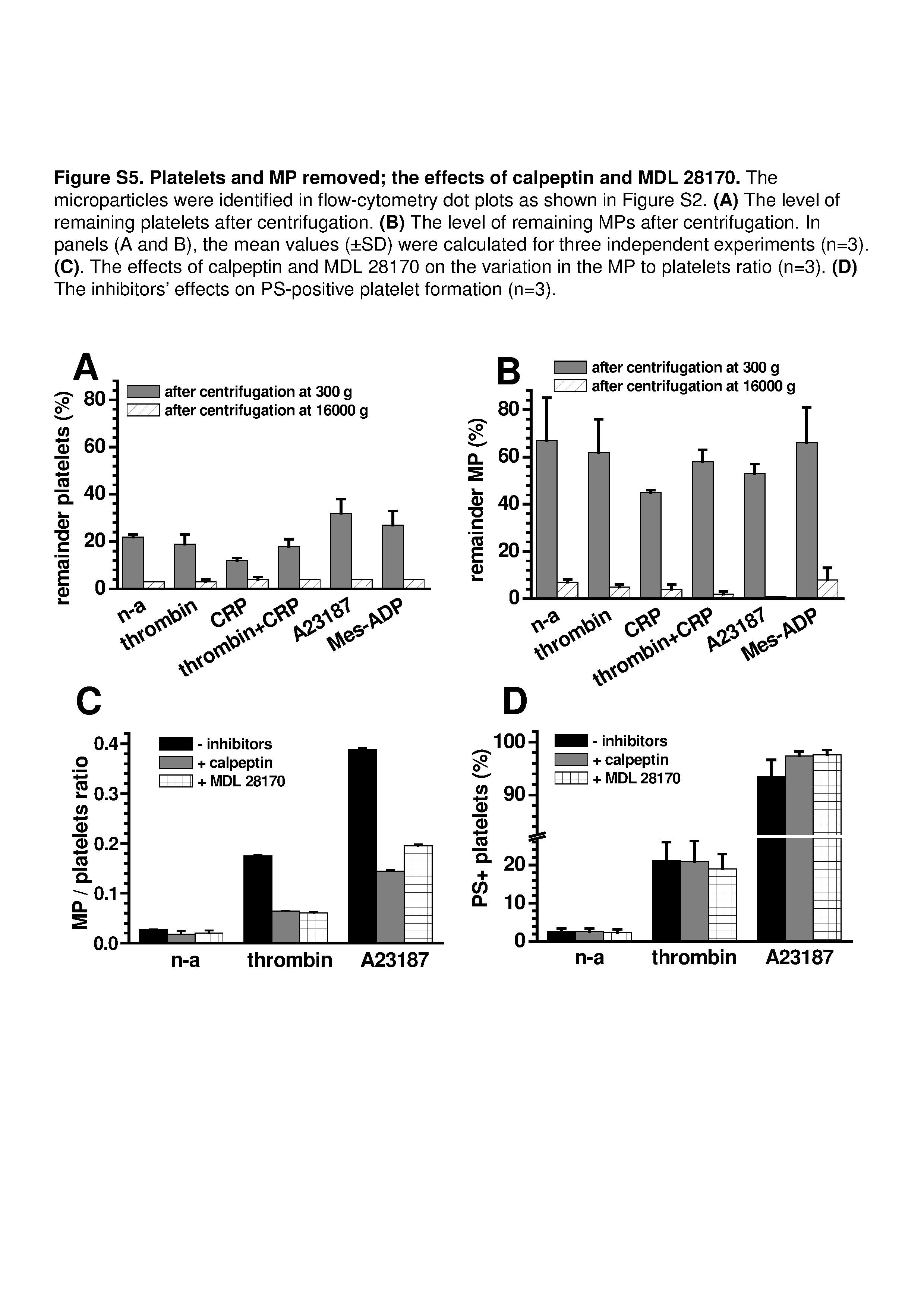

Supplement: S5 Fig — The microparticles were identified in flow-cytometry dot plots as shown in S2 Fig. (A) The level of remaining platelets after centrifugation. (B) The level of remaining MPs after centrifugation. In panels (A and B), the mean values (±SD) were calculated for three independent experiments (n = 3). (C). The effects of calpeptin and MDL 28170 on the variation in the MP to platelets ratio (n = 3). (D) The inhibitors’ effects on PS-positive platelet formation (n = 3). (TIF) [file pone.0116665.s005.tif]

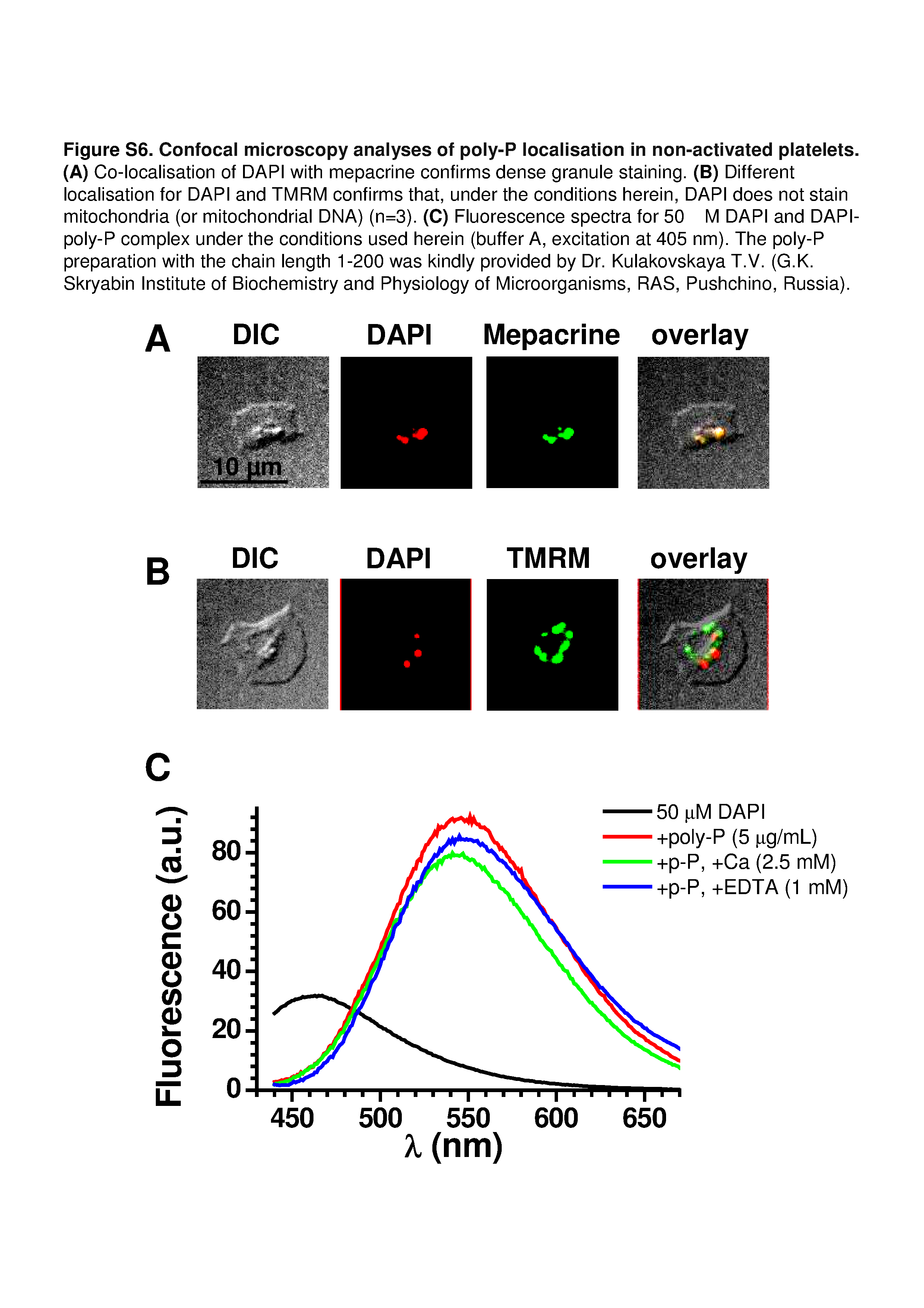

Supplement: S6 Fig — (A) Co-localisation of DAPI with mepacrine confirms dense granule staining. (B) Different localisation for DAPI and TMRM confirms that, under the conditions herein, DAPI does not stain mitochondria (or mitochondrial DNA) (n = 3). (C) Fluorescence spectra for 50 mM DAPI and DAPI-poly-P complex under the conditions used herein (buffer A, excitation at 405 nm). The poly-P preparation with the chain length 1–200 was kindly provided by Dr. Kulakovskaya T.V. (G.K. Skryabin Institute of Biochemistry and Physiology of Microorganisms, RAS, Pushchino, Russia). (TIF) [file pone.0116665.s006.tif]

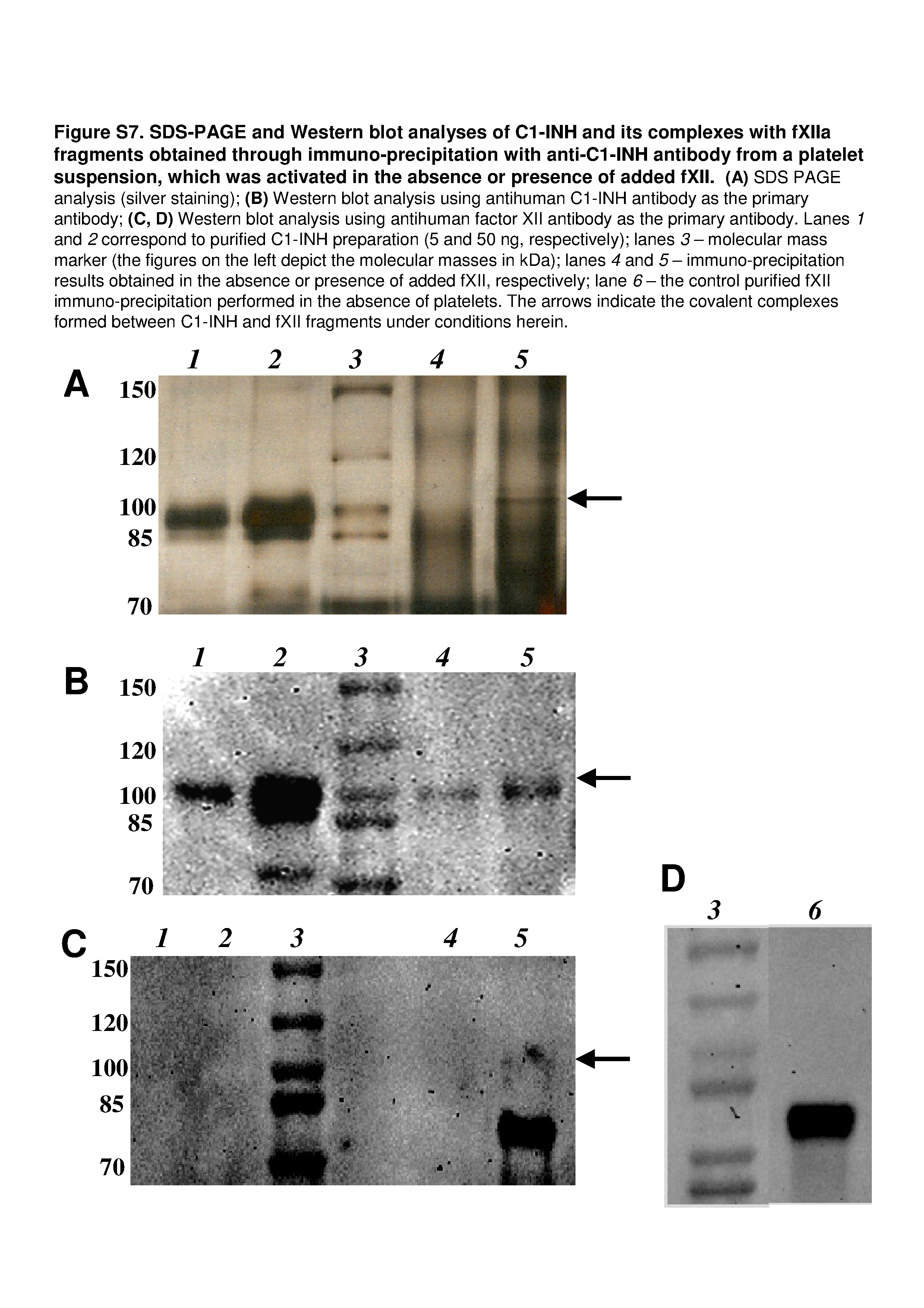

Supplement: S7 Fig — (A) SDS PAGE analysis (silver staining); (B) Western blot analysis using antihuman C1-INH antibody as the primary antibody; (C, D) Western blot analysis using antihuman factor XII antibody as the primary antibody. Lanes 1 and 2 correspond to purified C1-INH preparation (5 and 50 ng, respectively); lanes 3—molecular mass marker (the figures on the left depict the molecular masses in kDa); lanes 4 and 5—immuno-precipitation results obtained in the absence or presence of added fXII, respectively; lane 6—the control purified fXII immuno-precipitation performed in the absence of platelets. The arrows indicate the covalent complexes formed between C1-INH and fXII fragments under conditions herein. (TIF) [file pone.0116665.s007.tif]
